# Comprehensive Analysis of the Butyrate-Metabolism-Related Gene Signature in Tumor Microenvironment-Infiltrating Immune Cells in Clear Cell Renal Cell Carcinoma

**DOI:** 10.3389/fcell.2022.816024

**Published:** 2022-05-19

**Authors:** Gang Tang, Haochen Guan, Zhiyong Du, Weijie Yuan

**Affiliations:** ^1^ Department of Nephrology, Shanghai General Hospital, Shanghai Jiao Tong University School of Medicine, Shanghai, China; ^2^ Department of Urology Surgery, Dingzhou People’s Hospital of Hebei Province, Dingzhou, China

**Keywords:** butyrate, metabolism, tumor immune microenvironment, ccRCC, gene signature

## Abstract

A wealth of experimental evidence has validated that butyrate is capable of inhibiting tumorigenesis, while the potential role of butyrate metabolism in the tumor immune microenvironment (TIME) has been rarely explored. This study aims to explore the potential of butyrate-metabolism-related genes as prognostic biomarkers and their correlations with immune infiltrates in clear cell renal cell carcinoma (ccRCC) patients. Based on The Cancer Genome Atlas dataset (TCGA; *n* = 539), a total of 22 differentially expressed genes (DEGs) related with butyrate metabolism in ccRCC and normal samples were identified. Among them, a prognostic signature involving six butyrate-metabolism-related genes was created (Bu-Meta-GPS) in the training set (*n* = 271) and validation set (*n* = 268), and risk scores were calculated based on them. ccRCC patients with high-risk scores exhibited an unfavorable prognosis, high immunoscore, upregulated immuno-oncological targets (*PD1*, *PD-L1*, *CTLA4*, and *CD19*), and distinct immune-cell infiltration than those with low-risk scores. High-risk ccRCC patients without radiotherapy had a better survival rate than radiotherapy-treated patients. The negative regulation of cytokine production and cytokine-mediated signaling pathways was remarkably enriched in ccRCC patients with high-risk scores. A nomogram was then formulated to assess the overall survival (OS) of ccRCC patients. In summary, we illuminated the key role of butyrate metabolism in ccRCC TIME. The developed Bu-Meta-GPS was a sensitive predictive biomarker for the prognosis of ccRCC, which also provided new perspectives in improving immunotherapeutic efficacy.

## Introduction

Renal cell cancer (RCC) has multiple subtypes, and among them, 70% of them belong to ccRCC (Jonasch et al., 2021). Early-stage ccRCC usually presents an acceptable outcome following surgery or ablation. However, one-third of ccRCC patients suffer metastases, which are highly lethal and different from non-metastatic lesions (Jonasch et al., 2014; Jonasch et al., 2021). So far, large-scale clinical trials on ccRCC have been conducted, although its OS has not been remarkably improved by any systemic adjuvant therapy (Ravaud, 2017; Gross-Goupil et al., 2018). Stromal infiltration, immune infiltration, and transformed cells constitute the complex environment of the tumor. Depending on the type of cancer or tumor model, tumor-infiltrating cells are capable of indicating tumor suppression or promotion based on the cancer type (Senbabaoglu et al., 2016). Previous evidence demonstrated that ccRCC is a highly immune-infiltrated tumor (Thompson et al.,2007; Yoshihara et al., 2013). ccRCC is also one of the earliest malignant tumors responsive to immunotherapy in history and the most responsive one currently (Topalian et al., 2012; Herbst et al., 2014). About 1% of ccRCC patients present spontaneous regression, which is attributed to the immune mediation (Janiszewska et al., 2013), while the underlying mechanisms of spontaneous remission, immune infiltration, and immunotherapy response are poorly understood. The high tumor mutational burden has triggered a great breakthrough in the immune checkpoint blocking treatment of tumors (Snyder et al., 2014; Rizvi et al., 2015). It is expected that more tumor mutations will result in more MHC-binding neoantigens that are responsible for mediating tumor immune infiltration and chemotherapy response (Igarashi et al., 2002; Gubin et al., 2014; Snyder et al., 2014; Yadav et al., 2014). Previous data have demonstrated exciting outcomes in metastatic ccRCC patients intervened with immune checkpoint inhibitors (Motzer et al., 2018; Ghatalia et al., 2019), and their potential benefits are being assessed. Therefore, it is of great significance to illustrate ccRCC TIME, which presents a potential prognostic value and assists decision-making of adjuvant therapy.

A growing amount of evidence has shown the close link between human gut microbiota and carcinoma via specific pathogens or a wide range of microbial communities, especially their metabolites (Louis et al., 2014). Butyrate is a typical metabolite with an anti-cancer role against inflammation and uncontrolled malignant behaviors of cancer cells as well as accelerating their apoptosis and differentiation (Andoh et al., 2002; Hinnebusch et al., 2002; Zeng and Briske-Anderson, 2005; Comalada et al., 2006; O'Keefe, 2016). A decreased fecal butyrate level serves as an indicator for cancer risk, progression, and severity (Hu et al., 2016). In addition, intracellular butyrate promotes hyperacetylation of histones by inhibiting histone deacetylase (HDAC) activities in cancer and immune cells, which also regulates proteins and transcription factors involved in key signaling pathways (Wilson et al.,2010; Fung et al., 2012). As a result, butyrate is able to mediate expression and differentiation of various genes and pro-inflammatory cytokines (Chang et al., 2014). A latest study revealed that butyrate performed anti-tumoral effects (Smith et al., 2013) by regulatory T cells inhibiting local inflammatory responses (Arpaia et al., 2013; Furusawa et al., 2013). Moreover, extracellular butyrate may interact with surface-exposed G protein-coupled receptors (GPRs) in host cells. Serving as tumor suppressors, GPR43 and GPR109A are involved in the anti-cancer role of butyrate associated with a high fiber intake (Ganapathy et al., 2013). Butyrate is found to downregulate primary miR-17-92a and precursor and mature miR-92a in cancer cells (Hu et al., 2015), which is attributed to the inhibited proliferative and accelerated apoptotic rate via downregulating the oncogene MYC and upregulating CDKN1C (also known as p57), respectively (Hu et al., 2011).

Recent studies have found that butyrate directly enhances the *in vitro* and *in vivo* anti-cancer response of CD8^+^ T cells via activating the IL-12 signaling, indicating the anti-cancer therapy potential of butyrate (He et al., 2021). In the metastatic liver of mice with colorectal cancer, butyrate intervention positively mediates the section of IL-10 and IL-17 via T regulatory cells and natural killer T cells and T helper 17 cells, respectively (Ma et al., 2020). However, it is still not fully understood whether the butyrate could regulate immune-infiltrate and how it modulates immune response in ccRCC.

In this work, the prognostic value of the butyrate metabolism was assessed via bioinformatics in the ccRCC dataset downloaded from TCGA. The risk score was calculated to construct Bu-Meta-GPS based on a prognostic signature involving six identified genes strongly correlated with ccRCC prognosis. The influences of risk scores on immune-oncological targets (*PD1*, *CTLA4*, and *CD19*), immunoscore, and immune cell infiltration were then assessed, thus revealing the effect of the butyrate metabolism on ccRCC TIME. A nomogram was formulated, aiming to assess the prognosis of ccRCC patients with varying clinical characteristics. Our findings are expected to guide ccRCC immunotherapies in clinical practice.

## Materials and Methods

### ccRCC Dataset

RNA-seq data of 539 ccRCC patients and 348 butyrate-metabolism-related genes were obtained from TCGA (https://portal.gdc.cancer.gov/) and the Gene Set Enrichment Analysis (GSEA) database (https://www.gsea-msigdb.org/gsea/index.jsp), respectively, and the latter were identified based on available mRNA expression data of the former.

### Bioinformatics Analysis

DEGs related with butyrate metabolism between ccRCC and normal tissues were screened by the limma R package, with the false discovery rate controlled via *p*-value (FDR <0.05). The principal component analysis (PCA) was conducted using the R v4.1.0 package to identify gene-expression patterns between ccRCC and normal tissues. The tumor mutation burden (TMB) and somatic cell copy number alternations (CNAs) were calculated to determine the genomic variance.

Bu-Meta-GPS in the training set was created using the least absolute shrinkage and selection operator (LASSO) regression. The top six DEGs were selected for the following analysis. Risk scores were calculated according to expression levels and weighted coefficients obtained from the LASSO regression algorithm as follows: risk score = sum of coefficients × expression levels of six DEGs. High and low risks of ccRCC in the training and validation sets were classified using the median risk score as the cut-off.

ESTIMATE in R was adopted to estimate the immunoscore, stromal score, and ESTIMATE score-based prognostic signature in ccRCC TIME. Using the CIBERSORTx tool (https://cibersort.stanford.edu/), the infiltration levels of 22 types of immune cells were estimated, and those with CIBERSORT *p*-values of less than 0.05 were screened out for further analysis.

Java-based GSEA was performed to identify the survival difference among ccRCC subtypes. Gmt files (go.v7.4. symbols.gmt) were analyzed as the reference gene set in the MSigDB database. A random permutation of 1,000 was conducted. Pathways with *p* < 0.05 and a normalized enrichment score (NES) were considered significantly enriched.

### Statistical Analysis

Expression levels of butyrate-metabolism-related genes in ccRCC and normal tissues were examined using the Mann–Whitney *U*-test. Differences between groups and among multiple groups were compared using Student’s *t-*test and one-way ANOVA, respectively. Kaplan–Meier curves were depicted to compare OS between groups. Pearson’s correlation test was conducted to assess the correlation between risk scores, clinical features, immune-oncological targets, and immune infiltration levels. The independent prognostic potential of Bu-Meta-GPS was assessed by the univariate and multivariate Cox regression analyses. Receiver operating characteristic (ROC) curves and the area under the curve (AUC) were introduced to assess the prognostic value. A nomogram based on multivariate Cox regression analysis was created to determine the accuracy in predicting the prognosis. A significant difference was set at *p* < 0.05, which indicated statistical significance. GraphPad Prism 7.0 and R v. 4.1.0 were used to perform statistical analyses.

## Results

### Expression Levels of Butyrate-Metabolism-Related Genes in ccRCC

**FIGURE 1:**
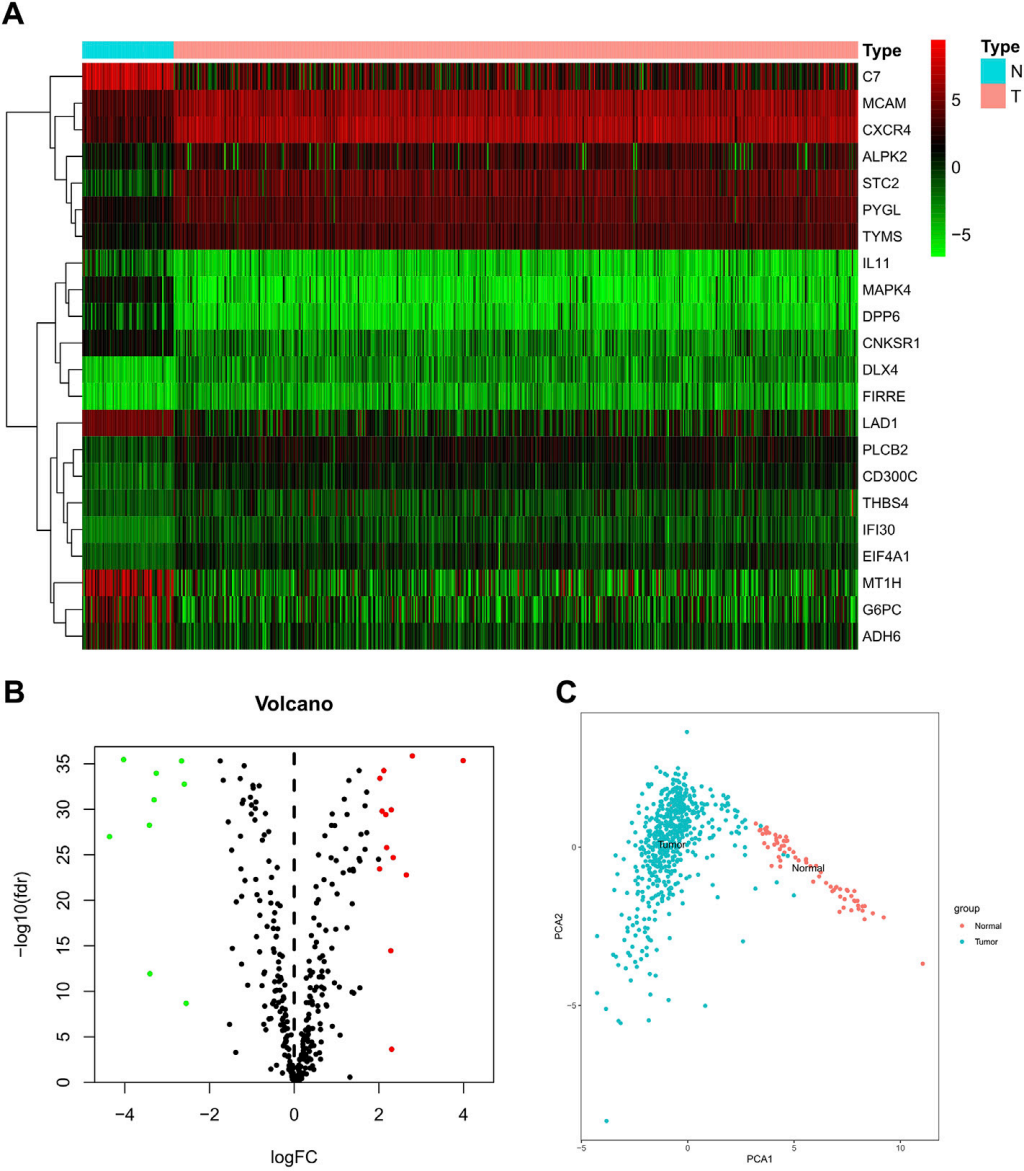
Different expressions of butyrate-metabolism-related genes in ccRCC and normal renal tissues. **(A)** Heatmap for 22 butyrate-metabolism-related genes from TCGA-KIRC cohort. **(B)** Volcano plot for screened butyrate-metabolism-related genes. **(C)** Principal component analysis for the expression of butyrate-metabolism-related genes to distinguish tumors (*n* = 539) from normal samples (*n* = 79) in TCGA cohort.

A dataset involving 539 ccRCC samples and 79 adjacent normal ones was downloaded from TCGA. Differentially expressed butyrate-metabolism-related genes were identified, and finally, 22 genes were screened out for further explorations (Figures 1A,B). To characterize the heterogeneity, PCA was performed to analyze mRNA levels of 22 butyrate-metabolism-related genes, which presented a significant difference (Figure 1C). The aforementioned analysis indicated the potential effect of butyrate-metabolism-related genes on distinguishing normal samples from ccRCC.

### Genomic Variance of Butyrate-Metabolism-Related Genes in ccRCC

**FIGURE 2:**
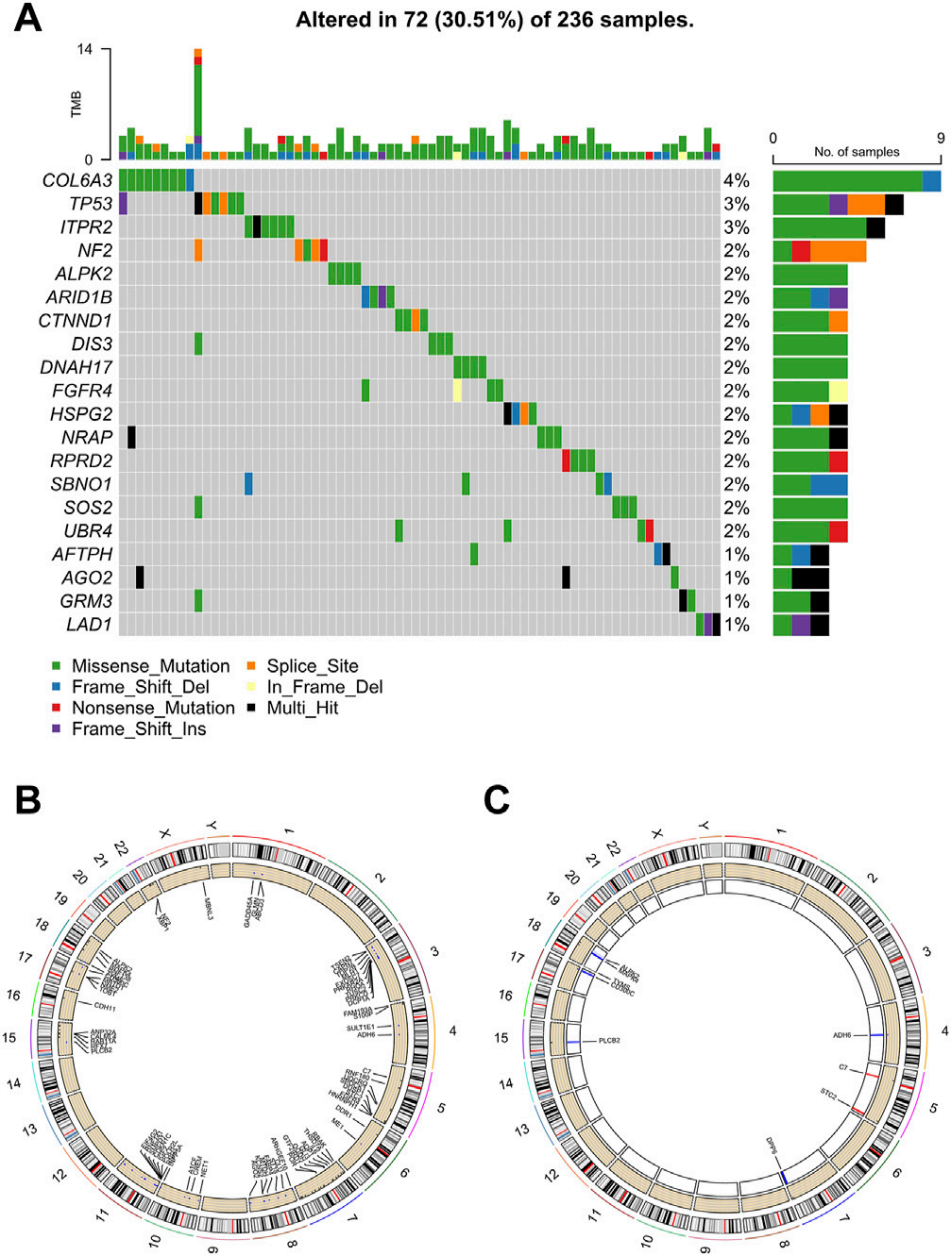
Characteristics of butyrate-metabolism-related genes in ccRCC. **(A)** Landscape of mutation profiles in 236 ccRCC patients from TCGA-KIRC cohort. **(B)** Location of CNV alteration of the butyrate-metabolism-related genes on chromosomes. **(C)** Location of CNV alteration of the DEGs on chromosomes.

A landscape of the incidence of somatic mutations of butyrate-metabolism-related genes in ccRCC patients from TCGA dataset was obtained. In the available samples, 72/236 (30.51%) ccRCC patients experienced mutations of butyrate-metabolism-related genes (top 20 genes were shown), with a frequency ranging from 1 to 4% (Figure 2A). Among them, the highest number of mutations was detected in the *COL6A3* gene (4%). In addition, the missense mutation was the most detected variant classification, and the main variant type was single-nucleotide polymorphism (SNP) (Supplementary Figure S1). Copy number variations (CNVs) were detected in 97/348 (27.87%) genes, and most of them were copy number amplifications (Figure 2B). Notably, among the 22 DEGs, nine genes (40.91%) had CNVs, including *ADH6*, *C7*, *STC2*, *DPP6*, *PLCB2*, *CD300C*, *TYMS*, *MAPK4*, and *ALPK2* (Figure 2C). Thus, it is believed that CNVs may be responsible for DEGs in ccRCC.

### Construction and Validation of Bu-Meta-GPS in ccRCC

A total of 539 ccRCC patients in TCGA dataset were randomly assigned to the training set (*n* = 271) and validation set (*n* = 268). LASSO regression analysis identified 6/22 DEGs in the training set, which were used to create Bu-Meta-GPS. Risk scores in both sets were then calculated by the following equation: Risk scores = 0.7145 × *DLX4* level +0.0439 ×*PLCB2* level +0.8463 ×*FIRRE* level +0.0752 × *IL-11* level -0.0178 × *ADH6* level +0.0026 × *EIF4A1* level.

**FIGURE 3:**
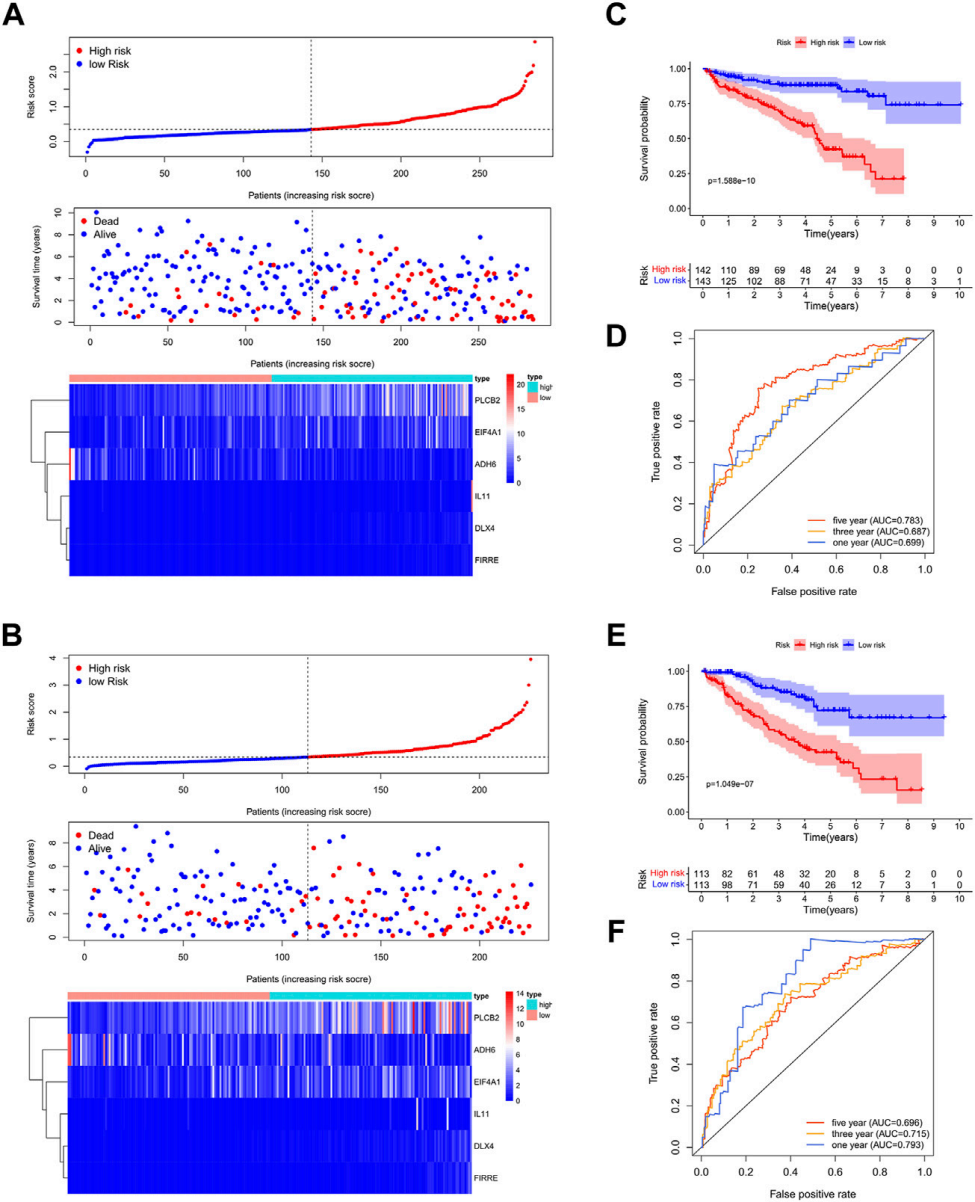
Construction and validation of prognostic signatures of butyrate-metabolism-related genes. Distribution of risk score, OS, and OS status and the heatmap of the six prognostic butyrate-metabolism-related genes in TCGA training cohort **(A)** and TCGA validation cohort **(B)**. Kaplan–Meier curves of OS for patients with ccRCC based on the risk score in TCGA training cohort **(C)** and TCGA validation cohort. **(E)** 1-, 3-, and 5-year ROC curves and AUC values in TCGA training cohort **(D)** and TCGA validation cohort **(F)**.

ccRCC patients were further subgrouped as high-risk and low-risk groups. The risk score distribution, OS, and Bu-Meta-GPS signature in both training and validation sets are depicted in Figures 3A,B. Low-risk patients had a significantly longer OS than high-risk patients in both sets (*p* < 0.001, Figures 3C,E). To further evaluate the prognostic accuracy of the Bu-Meta-GPS, 1-year, 3-year, and 5-year ROC curves were depicted, and the corresponding AUCs were calculated. It is shown that the 1-, 3-, and 5-year AUC values of the Bu-Meta-GPS in the training set were 0.699, 0.687, and 0.783, and those for the validation set were 0.793, 0.715, and 0.696 , respectively (Figures 3D,F). These findings indicated that the Bu-Meta-GPS involving butyrate-metabolism-related genes was a favorable prognostic tool for ccRCC.

### Independent Prognostic Value of Bu-Meta-GPS and Construction of the Bu-Meta-GPS-Based Nomogram

**FIGURE 4:**
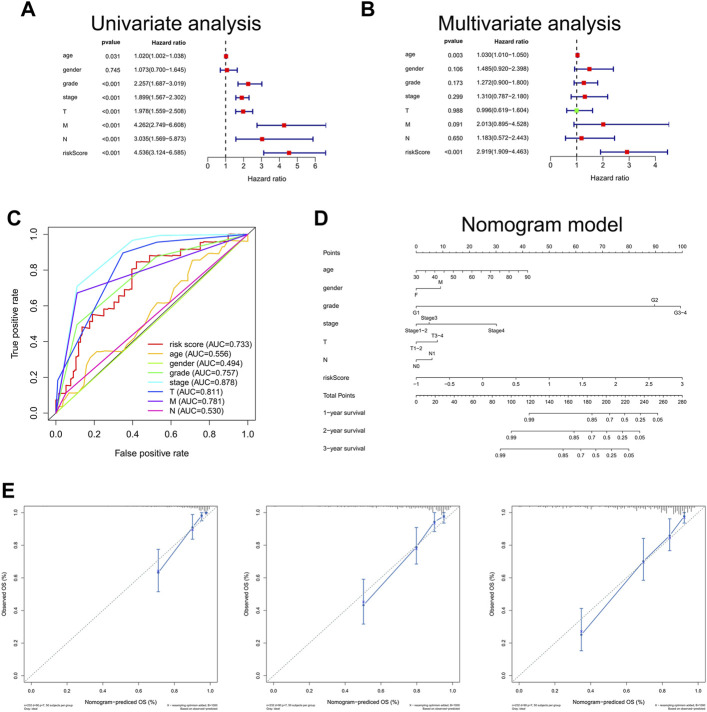
Independent prognostic role of risk scores. Univariate **(A)** and multivariate **(B)** Cox regression analyses for risk scores. **(C)** ROC curves and AUC values for risk score, age, gender, grade, stage, T, M, and N. **(D)** Nomogram based on risk score, age, gender, grade, stage, T, and N. **(E)** Calibration plots of the nomogram for predicting the probability of OS at 1, 2, and 3 years.

The univariate Cox regression analysis illustrated that the risk scores calculated by DEGs related with butyrate metabolism were correlated with OS of ccRCC patients (HR, 4.536; 95%CI, 3.124–6.585; *p* < 0.001; Figure 4A). Furthermore, Bu-Meta-GPS was validated by multivariate Cox regression analysis as an independent prognostic factor for ccRCC (HR, 2.919; 95%CI, 1.909–4.463; *p* < 0.001; Figure 4B). The AUC value for the Bu-Meta-GPS was 0.733, which was higher than that for the age (0.556), gender (0.494), and N stage (0.530) (Figure 4C).

A Bu-Meta-GPS-based nomogram was then created to test the 1-, 2-, and 3-year OS using risk scores and other clinical factors like the age, gender, grade, stage, and T and N stages (Figure 4D), which were consistent with the ones predicted by the nomogram calibration curves (Figure 4E).

### Bu-Meta-GPS Correlated With Clinical Features and Radiotherapy in ccRCC

**FIGURE 5:**
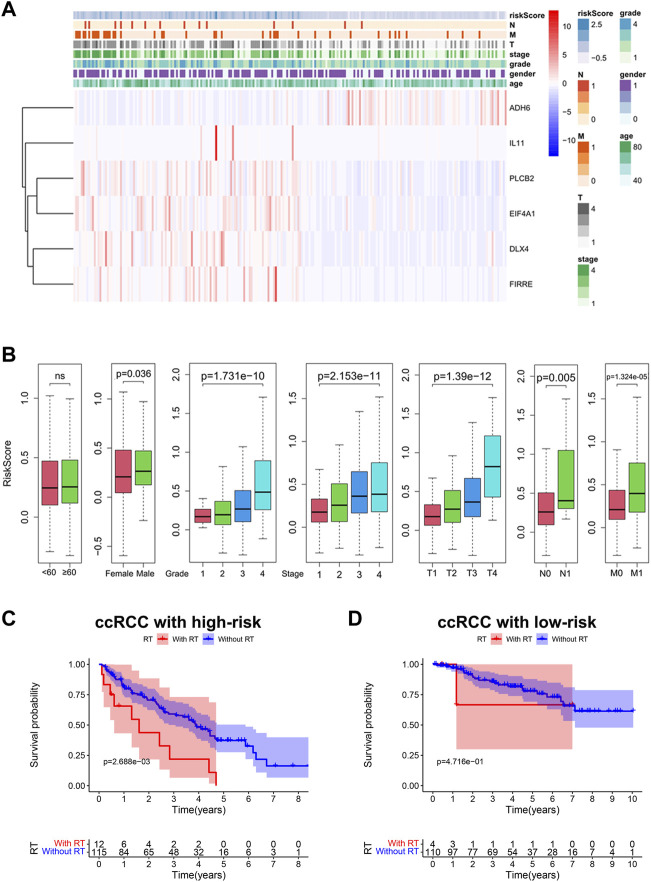
Bu-Meta-GPS correlated with clinical features and radiotherapy. **(A)** Heatmap of the association between the expression levels of the Bu-Meta-GPS and clinicopathological features. **(B)** Correlation of risk scores with age, gender, grade, stage, T, M, and N. Kaplan–Meier curves of OS for ccRCC patients subjected to radiotherapy in the high-risk group **(C)** and low-risk group **(D)** (ns, not statistically significant).

We further estimated the influence of risk scores on clinical features of ccRCC. Expression levels of the Bu-Meta-GPS classified by the risk level of the training set were depicted in a heatmap (Figure 5A). *IL-11*, *PLCB2*, *EIF4A1*, *DLX4*, and *FIRRE* were remarkably upregulated in high-risk patients than those of the other group, while *ADH6* was downregulated. We further examined the correlation of Bu-Meta-GPS with gender, age, stage, grade, and TNM staging in ccRCC patients, which revealed that the risk scores were positively correlated with tumor grade, stage, and TNM staging (*p* < 0.05, Figure 5B). Compared to female patients, male patients had high risk scores (*p* = 0.036, Figure 5B). It is suggested that Bu-Meta-GPS was significantly correlated with clinical features in ccRCC patients.

We next investigated the potential influence of risk scores on the prognostic performance in ccRCC patients subjected to radiotherapy. The results showed that high-risk ccRCC patients who did not receive radiotherapy had a better OS (Figure 5C). Although we did not obtain a significant difference in the survival benefit of radiotherapy in low-risk patients, it remained similar to that of the high-risk group (Figure 5D). Accordingly, high-risk ccRCC patients without radiotherapy had a better survival rate than radiotherapy-treated patients.

### Correlation Between Bu-Meta-GPS and Distinct Immune Cell Infiltration in ccRCC

**FIGURE 6:**
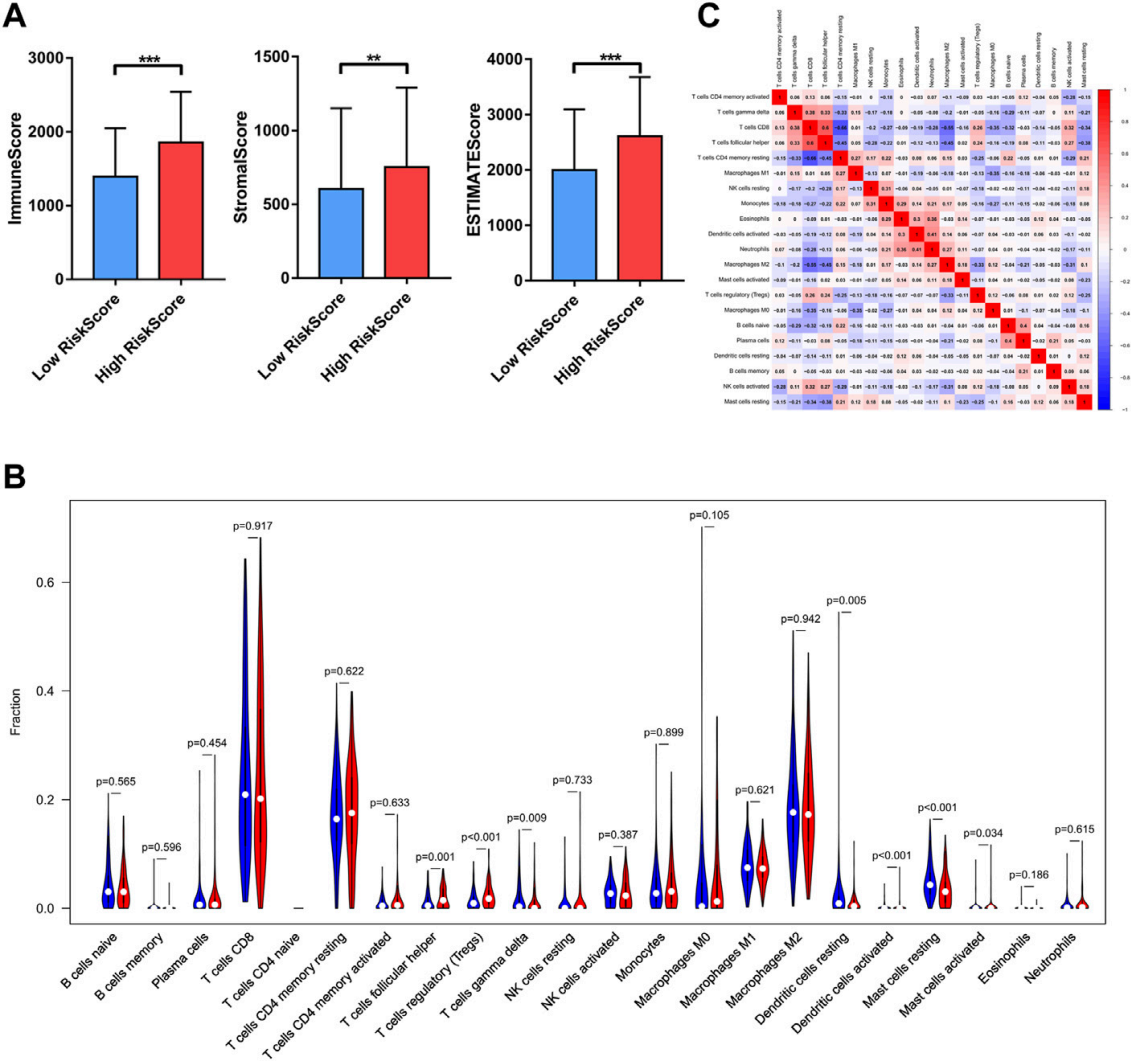
Relationship between Bu-Meta-GPS and immune cell infiltration. **(A)** Immune scores, stromal score, and ESTIMATE score in high- and low-risk groups. **(B)** Infiltrating levels of 22 immune cell types in high- and low-risk groups. **(C)** Correlation between the 22 immune cells (*p < 0.05; **p < 0.01; ***p < 0.001).

The difference in immune infiltrate levels classified by risk levels in ccRCC patients was examined, thus revealing the role of Bu-Meta-GPS in ccRCC TIME. Significantly higher immunoscore, stromal score, and ESTIMATE score were detected in the former (*p* < 0.01, Figure 6A). Subsequently, the fraction of 22 immune cell types between the two groups was analyzed. Higher infiltration levels of follicular helper T cells (Tfhs), regulatory T cells (Tregs), and activated mast cells were yielded in the high-risk group, while gamma delta T cells (γδ T cells), resting dendritic cells, and resting mast cells were mainly pronounced in the low-risk group (Figure 6B). In addition, we calculated the relationship between the immune cell types. A positive correlation was obtained between the number of Tfhs and CD8^+^ T cells (Figure 6C).

### Correlation Between Immuno-oncological Targets and Bu-Meta-GPS in ccRCC

**FIGURE 7:**
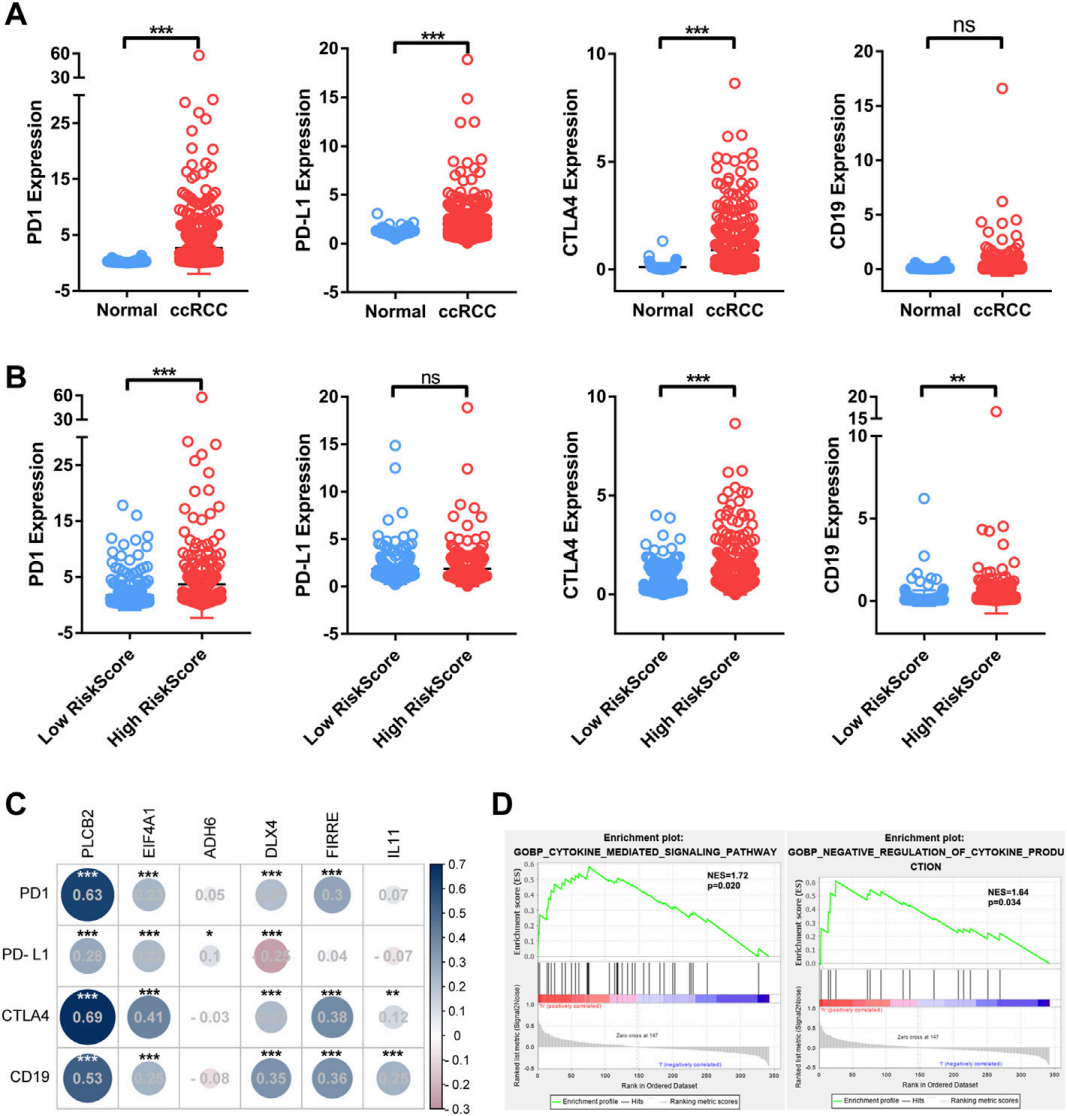
Association of four immuno-oncology targets with Bu-Meta-GPS in ccRCC. **(A)** Expression level of *PD-1*, *PD-L1*, *CTLA4*, and *CD19* in ccRCC tissues and normal renal tissues. **(B)** Expression level of *PD-1*, *PD-L1*, *CTLA4*, and *CD19* in high- and low-risk score groups. **(C)** Correlation of Bu-Meta-GPS with *PD-1*, *PD-L1*, *CTLA4*, and *CD19* in ccRCC. **(D)** GSEA showed that the cytokine-mediated signaling pathway was differentially enriched in the high-risk group (NES, normalized ES; *p < 0.05; **p < 0.01; ***p < 0.001; ns, not statistically significant).

To assess the correlation between immuno-oncological targets and Bu-Meta-GPS, their expression levels which differed from ccRCC risk were examined. *PD1*, *PD-L1*, and *CTLA4* were remarkably upregulated in ccRCC tissues than those of controls (*p* < 0.01; Figure 7A). Compared with those in low-risk patients, *PD1*, *CTLA4*, and *CD19* were upregulated in the high-risk group (*p* < 0.01; Figure 7B). In addition, *PD1*, *CTLA4*, and *CD19* levels were positively correlated with those of *PLCB2*, *EIF4A1*, *DLX4*, and *FIRRE*. The *PD-L1* level was positively correlated with those of *PLCB2* and *EIF4A1* but negatively correlated with *DLX4* (Figure 7C). GSEA was performed to illustrate the mechanism underlying the differences in the TIME and immuno-oncological targets between groups. It is shown that the cytokine-mediated signaling pathway and negative regulation of cytokine production were remarkably correlated with high-risk ccRCC patients (NES = 1.72 and 1.64; normalized *p* < 0.020 and 0.034, respectively; Figure 7D). Therefore, the cytokine-mediated signaling pathway may be attributed to different TIMEs and immuno-oncological targets classified by ccRCC risk.

## Discussion

Previous evidence has shown the anti-cancer effect of butyrate on multiple types of tumor cells via varying mechanisms (O'Keefe, 2016). It induces mitochondrial fusion and apoptosis in colorectal cancer cells via DRP1 (Tailor et al., 2014). Butyrate also inhibits migration of bladder cancer cells via inducing autophagy and apoptosis (Wang et al., 2020). Additionally, butyrate triggers apoptosis by overproducing reactive oxygen species (ROS) and causing mitochondrial dysfunction in breast cancer cells (Salimi et al., 2017). Multiple functions of butyrate in cancers indicate an extremely complicated regulatory mechanism. At present, cancer-intrinsic oncogene pathways have been well concerned. To clarify the potential regulatory mechanism of butyrate metabolism in TIME, it is necessary to fully analyze the function of butyrate. So far, the research on butyrate metabolism in ccRCC TIME is scant.

We have identified 348 DEGs related with butyrate metabolism and their prognostic values and effects on ccRCC TIME, which were capable of distinguishing tumor tissues from normal tissues. Among them, expression levels of 22 genes were significantly altered, including upregulated *MCAM*, *CXCR4*, *ALPK2*, *STC2*, *PYGL*, *TYMS*, *DLX4*, *FIRRE*, *PLCB2*, *CD300C*, *THBS4*, *IFI30*, and *EIF4A1* and downregulated *C7*, *IL-11*, *MAPK4*, *DPP6*, *CNKSR1*, *LAD1*, *MT1H*, *G6PC*, and *ADH6*, in ccRCC samples. Solid evidence suggested that these 22 genes functioned either as oncogenes or tumor-suppressor genes in various types of tumors. It is concluded that dysregulated genes related with butyrate metabolism may be important in ccRCC.

The prognostic value of Bu-Meta-GPS involving six DEGs related with butyrate metabolism in ccRCC patients was assessed and then validated, which effectively stratified the risk level of ccRCC. Notably, high-risk ccRCC patients in the training and validation sets had a worse OS than those of the other group. Bu-Meta-GPS was also identified to be correlated with clinical features of ccRCC. The risk scores calculated by Bu-Meta-GPS were positively correlated with the grade, stage, and TNM in ccRCC patients, which was consistent with the finding of a worse prognosis in high-risk patients. Furthermore, the prognostic role of Bu-Meta-GPS in ccRCC was confirmed. Compared with clinicopathological features, Bu-Meta-GPS presented a superb accuracy in predicting the prognosis of ccRCC. In addition, a nomogram combining Bu-Meta-GPS and clinical features was created and validated as an effective tool to predict the survival of ccRCC. We found that Bu-Meta-GPS was also able to predict radiotherapy sensitivity in ccRCC patients, of whom patients with low-risk scores gained more benefits from radiotherapy. Current studies revealed that radiotherapy caused immunosuppressive effects in the tumor microenvironment due to the attraction of immunosuppressive cells such as regulatory T cells (Tregs) as well as because of the release of immunosuppressive cytokines (TGF-β and IL-10) and chemokines (Shevtsov et al., 2019; Sato et al., 2020). It is suggested that radiotherapy should be carefully considered for high-risk ccRCC patients.

In this study, the influence of Bu-Meta-GPS on ccRCC TIME was demonstrated. Risk scores of high-risk and low-risk ccRCC patients were correlated with distinct immune cell infiltration levels, immunoscore, and expression levels of immuno-oncological targets. The stromal score and immunoscore were significantly higher in high-risk ccRCC patients, indicating that the high immune/stromal score patients had poorer survival outcomes. Classified by cell types, the infiltration levels of Tfhs, Tregs, and activated mast cells in high-risk ccRCC patients were higher than those in the low-risk group, while those of γδ T cells, dendritic cells, and resting mast cells presented opposite trends. We considered that the immunoscore and immune cell infiltration were the main causes for different survival in ccRCC patients stratified by risk levels. In addition, higher levels of *PD-1*, *CTLA4*, and *CD19* were detected in high-risk patients, while the *PD-L1* level was comparable. *PD-L1* was significantly upregulated in ccRCC tissues compared to that in controls. Previous studies have demonstrated vital functions of *CTLA4* and *PD-L1* in tumorigenesis, tumor immunity, and prognosis (Zheng et al., 2019; Liu et al., 2020). Differentially expressed *CD274* and *CD19* genes are reported to be associated with the prognosis of ccRCC (Zhou et al., 2020). Our study also found that risk scores were significantly correlated with expression levels of *PD-1*, *PD-L1*, *CTLA4*, and *CD19*, suggesting that the four immune checkpoints may be potential immunotherapy targets for ccRCC. Yukihiro et al. reported that butyrate ameliorates the development of colitis via inducing the differentiation of Tregs (Furusawa et al., 2013). Yang et al. (2020) showed that butyrate promotes IL-22 production in human CD4^+^ T cells. These findings suggested that butyrate metabolism mediated ccRCC TIME. GSEA results demonstrated that the cytokine-mediated signaling pathway was mainly enriched in high-risk ccRCC patients, suggesting that vital genes involved in this pathway may be vital targets for butyrate metabolism. Considering this, butyrate metabolism and cytokine-mediated signaling pathways were responsible for different ccRCC TIMEs between groups.

Several limitations in the present study should be noted. First, further verification failed due to a small sample size, which should be further validated in multi-center large-scale trials. Second, the regulatory mechanism of butyrate metabolism in ccRCC TIME requires an in-depth exploration.

In conclusion, we created a prognostic signature involving butyrate-metabolism-related genes and evaluated their influence on ccRCC TIME, which provided prognostic markers and immunotherapy targets for ccRCC.

## Data Availability

The datasets presented in this study can be found in online repositories. The names of the repository/repositories and accession number(s) can be found at: https://portal.gdc.cancer.gov/.

## References

[B1] Andoh A. Shimada M. Araki Y. Fujiyama Y. Bamba T. (2002). Sodium Butyrate Enhances Complement-Mediated Cell Injury via Down-Regulation of Decay-Accelerating Factor Expression in Colonic Cancer Cells. Cancer Immunol. Immunother. 50, 663–672. 10.1007/s00262-001-0239-1 11862418 PMC11033022

[B2] Arpaia N. Campbell C. Fan X. Dikiy S. van der Veeken J. deRoos P. (2013). Metabolites Produced by Commensal Bacteria Promote Peripheral Regulatory T-Cell Generation. Nature 504, 451–455. 10.1038/nature12726 24226773 PMC3869884

[B3] Chang P. V. Hao L. Offermanns S. Medzhitov R. (2014). The Microbial Metabolite Butyrate Regulates Intestinal Macrophage Function via Histone Deacetylase Inhibition. Proc. Natl. Acad. Sci. U.S.A. 111, 2247–2252. 10.1073/pnas.1322269111 24390544 PMC3926023

[B4] Comalada M. Bailón E. de Haro O. Lara-Villoslada F. Xaus J. Zarzuelo A. (2006). The Effects of Short-Chain Fatty Acids on colon Epithelial Proliferation and Survival Depend on the Cellular Phenotype. J. Cancer Res. Clin. Oncol. 132, 487–497. 10.1007/s00432-006-0092-x 16788843 PMC12161102

[B5] Fung K. Y. C. Cosgrove L. Lockett T. Head R. Topping D. L. (2012). A Review of the Potential Mechanisms for the Lowering of Colorectal Oncogenesis by Butyrate. Br. J. Nutr. 108, 820–831. 10.1017/S0007114512001948 22676885

[B6] Furusawa Y. Obata Y. Fukuda S. Endo T. A. Nakato G. Takahashi D. (2013). Commensal Microbe-Derived Butyrate Induces the Differentiation of Colonic Regulatory T Cells. Nature 504, 446–450. 10.1038/nature12721 24226770

[B7] Ganapathy V. Thangaraju M. Prasad P. D. Martin P. M. Singh N. (2013). Transporters and Receptors for Short-Chain Fatty Acids as the Molecular Link between Colonic Bacteria and the Host. Curr. Opin. Pharmacol. 13, 869–874. 10.1016/j.coph.2013.08.006 23978504

[B8] Ghatalia P. Gordetsky J. Kuo F. Dulaimi E. Cai K. Q. Devarajan K. (2019). Prognostic Impact of Immune Gene Expression Signature and Tumor Infiltrating Immune Cells in Localized clear Cell Renal Cell Carcinoma. J. Immunotherapy Cancer 7, 139. 10.1186/s40425-019-0621-1 PMC6540413 31138299

[B9] Gross-Goupil M. Kwon T. G. Eto M. Ye D. Miyake H. Seo S. I. (2018). Axitinib versus Placebo as an Adjuvant Treatment of Renal Cell Carcinoma: Results from the Phase III, Randomized ATLAS Trial. Ann. Oncol. 29, 2371–2378. 10.1093/annonc/mdy454 30346481 PMC6311952

[B10] Gubin M. M. Zhang X. Schuster H. Caron E. Ward J. P. Noguchi T. (2014). Checkpoint Blockade Cancer Immunotherapy Targets Tumour-specific Mutant Antigens. Nature 515, 577–581. 10.1038/nature13988 25428507 PMC4279952

[B11] He Y. Fu L. Li Y. Wang W. Gong M. Zhang J. (2021). Gut Microbial Metabolites Facilitate Anticancer Therapy Efficacy by Modulating Cytotoxic CD8+ T Cell Immunity. Cel Metab. 33, 988–1000. e7. 10.1016/j.cmet.2021.03.002 33761313

[B12] Herbst R. S. Soria J.-C. Kowanetz M. Fine G. D. Hamid O. Gordon M. S. (2014). Predictive Correlates of Response to the Anti-PD-L1 Antibody MPDL3280A in Cancer Patients. Nature 515, 563–567. 10.1038/nature14011 25428504 PMC4836193

[B13] Hinnebusch B. F. Meng S. Wu J. T. Archer S. Y. Hodin R. A. (2002). The Effects of Short-Chain Fatty Acids on Human colon Cancer Cell Phenotype Are Associated with Histone Hyperacetylation. J. Nutr. 132, 1012–1017. 10.1093/jn/132.5.1012 11983830

[B14] Hu S. Dong T. S. Dalal S. R. Wu F. Bissonnette M. Kwon J. H. (2011). The Microbe-Derived Short Chain Fatty Acid Butyrate Targets miRNA-dependent P21 Gene Expression in Human colon Cancer. PLoS One 6, e16221. 10.1371/journal.pone.0016221 21283757 PMC3024403

[B15] Hu S. Liu L. Chang E. B. Wang J.-Y. Raufman J.-P. (2015). Butyrate Inhibits Pro-proliferative miR-92a by Diminishing C-Myc-Induced miR-17-92a Cluster Transcription in Human colon Cancer Cells. Mol. Cancer 14, 180. 10.1186/s12943-015-0450-x 26463716 PMC4604099

[B16] Hu Y. Le Leu R. K. Christophersen C. T. Somashekar R. Conlon M. A. Meng X. Q. (2016). Manipulation of the Gut Microbiota Using Resistant Starch Is Associated with protection against Colitis-Associated Colorectal Cancer in Rats. Carcin 37, 366–375. 10.1093/carcin/bgw019 26905582

[B17] Igarashi T. Takahashi H. Tobe T. Suzuki H. Mizoguchi K. Nakatsu H.-o. (2002). Effect of Tumor-Infiltrating Lymphocyte Subsets on Prognosis and Susceptibility to Interferon Therapy in Patients with Renal Cell Carcinoma. Urol. Int. 69, 51–56. 10.1159/000064361 12119440

[B18] Janiszewska A. D. Poletajew S. Wasiutyński A. (2013). Reviews Spontaneous Regression of Renal Cell Carcinoma. wo 2, 123–127. 10.5114/wo.2013.34613 PMC3685371 23788977

[B19] Jonasch E. Gao J. Rathmell W. K. (2014). Renal Cell Carcinoma. BMJ 349, g4797. 10.1136/bmj.g4797 25385470 PMC4707715

[B20] Jonasch E. Walker C. L. Rathmell W. K. (2021). Clear Cell Renal Cell Carcinoma Ontogeny and Mechanisms of Lethality. Nat. Rev. Nephrol. 17, 245–261. 10.1038/s41581-020-00359-2 33144689 PMC8172121

[B21] Liu J.-N. Kong X.-S. Huang T. Wang R. Li W. Chen Q.-F. (2020). Clinical Implications of Aberrant PD-1 and CTLA4 Expression for Cancer Immunity and Prognosis: A Pan-Cancer Study. Front. Immunol. 11, 2048. 10.3389/fimmu.2020.02048 33072070 PMC7539667

[B22] Louis P. Hold G. L. Flint H. J. (2014). The Gut Microbiota, Bacterial Metabolites and Colorectal Cancer. Nat. Rev. Microbiol. 12, 661–672. 10.1038/nrmicro3344 25198138

[B23] Ma X. Zhou Z. Zhang X. Fan M. Hong Y. Feng Y. (2020). Sodium Butyrate Modulates Gut Microbiota and Immune Response in Colorectal Cancer Liver Metastatic Mice. Cell Biol Toxicol 36, 509–515. 10.1007/s10565-020-09518-4 32172331

[B24] Motzer R. J. Tannir N. M. McDermott D. F. Arén Frontera O. Melichar B. Choueiri T. K. (2018). Nivolumab Plus Ipilimumab versus Sunitinib in Advanced Renal-Cell Carcinoma. N. Engl. J. Med. 378, 1277–1290. 10.1056/NEJMoa1712126 29562145 PMC5972549

[B25] O'Keefe S. J. D. (2016). Diet, Microorganisms and Their Metabolites, and colon Cancer. Nat. Rev. Gastroenterol. Hepatol. 13, 691–706. 10.1038/nrgastro.2016.165 27848961 PMC6312102

[B26] Ravaud A. (2017). Adjuvant Sunitinib in Renal-Cell Carcinoma. N. Engl. J. Med. 376, 893. 10.1056/NEJMc1616636 28249133

[B27] Rizvi N. A. Hellmann M. D. Snyder A. Kvistborg P. Makarov V. Havel J. J. (2015). Mutational Landscape Determines Sensitivity to PD-1 Blockade in Non-small Cell Lung Cancer. Science 348, 124–128. 10.1126/science.aaa1348 25765070 PMC4993154

[B28] Salimi V. Shahsavari Z. Safizadeh B. Hosseini A. Khademian N. Tavakoli-Yaraki M. (2017). Sodium Butyrate Promotes Apoptosis in Breast Cancer Cells through Reactive Oxygen Species (ROS) Formation and Mitochondrial Impairment. Lipids Health Dis. 16, 208. 10.1186/s12944-017-0593-4 29096636 PMC5669027

[B29] Sato H. Okonogi N. Nakano T. (2020). Rationale of Combination of Anti-PD-1/pd-L1 Antibody Therapy and Radiotherapy for Cancer Treatment. Int. J. Clin. Oncol. 25, 801–809. 10.1007/s10147-020-01666-1 32246277 PMC7192886

[B30] Şenbabaoğlu Y. Gejman R. S. Winer A. G. Liu M. Van Allen E. M. de Velasco G. (2016). Tumor Immune Microenvironment Characterization in clear Cell Renal Cell Carcinoma Identifies Prognostic and Immunotherapeutically Relevant Messenger RNA Signatures. Genome Biol. 17, 231. 10.1186/s13059-016-1092-z 27855702 PMC5114739

[B31] Shevtsov M. Sato H. Multhoff G. Shibata A. (2019). Novel Approaches to Improve the Efficacy of Immuno-Radiotherapy. Front. Oncol. 9, 156. 10.3389/fonc.2019.00156 30941308 PMC6433964

[B32] Smith P. M. Howitt M. R. Panikov N. Michaud M. Gallini C. A. Bohlooly-Y M. (2013). The Microbial Metabolites, Short-Chain Fatty Acids, Regulate Colonic T Reg Cell Homeostasis. Science 341, 569–573. 10.1126/science.1241165 23828891 PMC3807819

[B33] Snyder A. Makarov V. Merghoub T. Yuan J. Zaretsky J. M. Desrichard A. (2014). Genetic Basis for Clinical Response to CTLA-4 Blockade in Melanoma. N. Engl. J. Med. 371, 2189–2199. 10.1056/NEJMoa1406498 25409260 PMC4315319

[B34] Tailor D. Hahm E.-R. Kale R. K. Singh S. V. Singh R. P. (2014). Sodium Butyrate Induces DRP1-Mediated Mitochondrial Fusion and Apoptosis in Human Colorectal Cancer Cells. Mitochondrion 16, 55–64. 10.1016/j.mito.2013.10.004 24177748 PMC4004730

[B35] Thompson R. H. Dong H. Lohse C. M. Leibovich B. C. Blute M. L. Cheville J. C. (2007). PD-1 Is Expressed by Tumor-Infiltrating Immune Cells and Is Associated with Poor Outcome for Patients with Renal Cell Carcinoma. Clin. Cancer Res. 13, 1757–1761. 10.1158/1078-0432.CCR-06-2599 17363529

[B36] Topalian S. L. Hodi F. S. Brahmer J. R. Gettinger S. N. Smith D. C. McDermott D. F. (2012). Safety, Activity, and Immune Correlates of Anti-PD-1 Antibody in Cancer. N. Engl. J. Med. 366, 2443–2454. 10.1056/NEJMoa1200690 22658127 PMC3544539

[B37] Wang F. Wu H. Fan M. Yu R. Zhang Y. Liu J. (2020). Sodium Butyrate Inhibits Migration and Induces AMPK‐mTOR Pathway‐dependent Autophagy and ROS‐mediated Apoptosis via the miR‐139‐5p/Bmi‐1 axis in Human Bladder Cancer Cells. FASEB j. 34, 4266–4282. 10.1096/fj.201902626R 31957111

[B38] Wilson A. J. Chueh A. C. Tögel L. Corner G. A. Ahmed N. Goel S. (2010). Apoptotic Sensitivity of colon Cancer Cells to Histone Deacetylase Inhibitors Is Mediated by an Sp1/Sp3-Activated Transcriptional Program Involving Immediate-Early Gene Induction. Cancer Res. 70, 609–620. 10.1158/0008-5472.CAN-09-2327 20068171 PMC2939837

[B39] Yadav M. Jhunjhunwala S. Phung Q. T. Lupardus P. Tanguay J. Bumbaca S. (2014). Predicting Immunogenic Tumour Mutations by Combining Mass Spectrometry and Exome Sequencing. Nature 515, 572–576. 10.1038/nature14001 25428506

[B40] Yang W. Yu T. Huang X. Bilotta A. J. Xu L. Lu Y. (2020). Intestinal Microbiota-Derived Short-Chain Fatty Acids Regulation of Immune Cell IL-22 Production and Gut Immunity. Nat. Commun. 11, 4457. 10.1038/s41467-020-18262-6 32901017 PMC7478978

[B41] Yoshihara K. Shahmoradgoli M. Martínez E. Vegesna R. Kim H. Torres-Garcia W. (2013). Inferring Tumour Purity and Stromal and Immune Cell Admixture from Expression Data. Nat. Commun. 4, 2612. 10.1038/ncomms3612 24113773 PMC3826632

[B42] Zeng H. Briske-Anderson M. (2005). Prolonged Butyrate Treatment Inhibits the Migration and Invasion Potential of HT1080 Tumor Cells. J. Nutr. 135, 291–295. 10.1093/jn/135.2.291 15671229

[B43] Zheng S. Zhang Z. Qu Y. Zhang X. Guo H. Shi X. (2019). Radiopharmaceuticals and Fluorescein Sodium Mediated Triple‐Modality Molecular Imaging Allows Precise Image‐Guided Tumor Surgery. Adv. Sci. 6, 1900159. 10.1002/advs.201900159 PMC6662088 31380183

[B44] Zhou Q.-H. Li K.-W. Chen X. He H.-X. Peng S.-M. Peng S.-R. (2020). HHLA2 and PD-L1 Co-expression Predicts Poor Prognosis in Patients with clear Cell Renal Cell Carcinoma. J. Immunother. Cancer 8, e000157. 10.1136/jitc-2019-000157 31959726 PMC7057441

