# Supplementary figures and images for: Comprehensive Analysis of the Butyrate-Metabolism-Related Gene Signature in Tumor Microenvironment-Infiltrating Immune Cells in Clear Cell Renal Cell Carcinoma
Source: Front Cell Dev Biol. 2022 May 19;10:816024. doi: 10.3389/fcell.2022.816024 (PMC9160722; doi:10.3389/fcell.2022.816024)

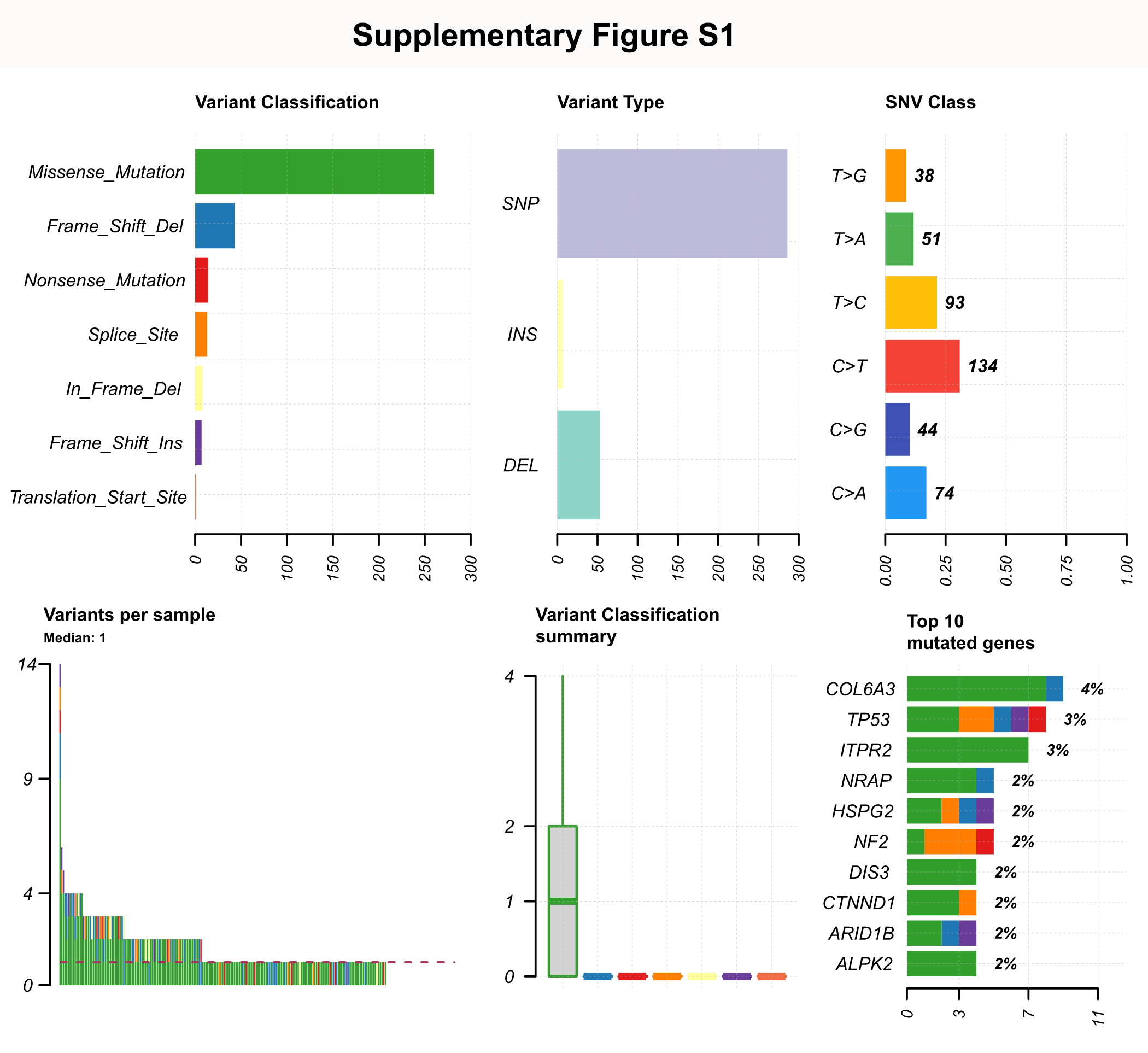

Supplement: Supplementary file 1 [file Image1.TIF]
